# Fuzzy Identity-Based Signature Scheme Suitable for Biometric Authentication

**DOI:** 10.3390/s26154896

**Published:** 2026-08-03

**Authors:** Yunyun Qu, Cuiju Ke, Songlin Tian, Miaomiao Yang, Na Wang

**Affiliations:** 1 School of Mathematical Sciences, Guizhou Normal University, Gui’an New District 550025, China; kcj@gznu.edu.cn (C.K.); tiansonglin@gznu.edu.cn (S.T.); ymm@gznu.edu.cn (M.Y.); 2 Guizhou Provincial Specialized Key Laboratory of Information Security Technology in Higher Education Institutions, Guizhou Normal University, Gui’an New District 550025, China; 3 Guizhou Key Laboratory of NewGen Cyberspace Security, Guiyang 550001, China; 4 School of Cyber Science and Technology, Beihang University, Beijing 100191, China; nawang@buaa.edu.cn

**Keywords:** standard model, computational Diffie–Hellman problem, bilinear pairings, fuzzy identity-based signature

## Abstract

The security of a signature scheme given in the standard model (SM) will be more stable and reliable than that given in the random oracle model (ROM). Fuzzy identity-based signature (FIBS) enables a user to generate a signature for a set of descriptive attributes, defined as $\omega ={\left\{\omega_{j}\right\}}_{j=1}^{n}$. Any attributes set $\omega^{\prime }={\left\{\omega_{j}^{\prime }\right\}}_{j=1}^{n}$ can validate the signature provided that the distance between ω and $\omega^{\prime }$ is below a predefined threshold. Most of the existing FIBS schemes are based on the ROM. It is of great significance to design a FIBS scheme based on the SM. In this work, we adopt fingerprint minutiae as the biometric modality and present a feature extraction algorithm E that transforms raw minutiae into quantized, privacy-preserving attribute sets, and we present a False Rejection Rate (FRR)–False Acceptance Rate (FAR) trade-off framework to calibrate matching threshold *t*, with adjustable *n* for qualified error performance. Subsequently, we present a novel and efficient FIBS scheme, which is proven to be unforgeable in SM for any polynomially bounded adversary under selective identity attack model. Compared to the existing FIBS schemes based on the ROM, our new FIBS scheme has a strong security model. Compared to the existing FIBS scheme based on the SM, our new FIBS scheme reduces total computation consumption by approximately 33.55% and achieves a significant reduction in communication consumption, saving approximately 68.07% of the message and signature size, which is suitable for biometric authentication.

## 1. Introduction

In 1985, Shamir [1] first proposed the concept of identity-based public key cryptography (ID-PKC), wherein an entity’s public key could be easily derived from his unique identity attributes (e.g., an e-mail address, IP address, etc.). In this system, the corresponding private key for each entity is generated by combining the identity information with a master secret key held by a trusted third party, known as the Private Key Generator (PKG). This cryptosystem subverts the traditional public key infrastructure’s practice of relying on certificates to authenticate public keys and solves the problem of certificate management. With the deep integration of blockchain and the Industrial Internet of Things (IIoT), decentralized identity (DID) and data provenance verification have become core security supports for intelligent flexible manufacturing. In 2024, Deng et al. [2] proposed FutureDID, a robust and practical decentralized identity (DID) system that enables multiple parties to jointly issue credentials and efficiently revoke DID identities. In 2025, Xu et al. [3] proposed a blockchain-based verifiable decentralized identity (DID) system for Industrial Internet of Things (IIoT). In 2026, Deng et al. [4] proposed vProChain, an efficient provenance verification system that supports verifiable and parallel queries over graph-structured provenance data. While the aforementioned schemes leverage blockchain for decentralized identity management, another line of research focuses on cryptographic primitives that tolerate identity errors or variations—an inherent challenge in real-world identity representation. To address this issue, early foundational work introduced the concept of fuzzy identity-based cryptography, which relaxes the exact-match requirement in traditional identity-based settings.

**Figure 1:**
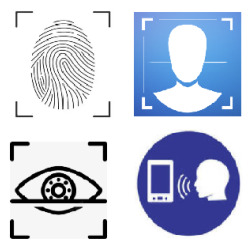
Biometric information.

Password-based authentication relies on simple passwords, which are vulnerable to dictionary attacks. Hence, it is suitable only for systems with lower security levels. Biometric information (such as fingerprints, face, iris and voice; see Figure 1) is extremely difficult to replicate, distribute, share and forge, and will never be forgotten or lost by people. Using biometric features such as fingerprints, facial features, iris and voice as identities, the identities have uniqueness. Biometric authentication based on biometric information is more reliable than password-based authentication and is suitable for application in systems with higher security levels. However, due to the noise in biometric measurements, fault-tolerant characteristics (fuzzy) can be used to construct cryptographic schemes such as encryption and signature based on biometric identity. In 2005, Sahai and Waters [5] first proposed the concept of fuzzy identity-based encryption (FIBE), wherein an entity’s identity is represented as a set of descriptive attributes rather than a simple string of characters. Within this FIBE framework, a user holding the secret key for an identity ID characterized by an attributes set ω can decrypt a ciphertext encrypted with the public key for another identity $ID^{\prime }$ characterized by an attributes set $\omega^{\prime }$, provided that ω and $\omega^{\prime }$ exhibit a predefined proximity. Consequently, the FIBE scheme permits some flexibility in identity mismatch tolerance. In 2012, Agrawal et al. [6] constructed fuzzy identity-based encryption based on the hardness of the learning with errors (LWE) problem. In 2024, Liu et al. [7] presented two new fault-tolerant identity-based encryption schemes based on the framework of SM9.

### 1.1. Related Work

In 2008, Yang et al. [8] originally proposed the notion of a fuzzy identity-based signature (FIBS) and developed a FIBS scheme construction derived from Sahai and Waters’ FIBE scheme. FIBS allows a user with an identity ID characterized by an attributes set $\omega ={\left\{\omega_{j}\right\}}_{j=1}^{n}$ to generate a signature that can be successfully verified against another identity $ID^{\prime }$ characterized by an attributes set $\omega^{\prime }={\left\{\omega_{j}^{\prime }\right\}}_{j=1}^{n}$, provided the sets satisfy the condition $|\omega \cap \omega^{\prime }|\;\geq t$, where *t* serves as an error tolerance threshold parameter. Consider a smartphone-based mobile payment system where a user signs a transaction using their fingerprint. In practice, due to sensor noise (e.g., capacitive sensing deviations, optical lens contamination), skin conditions (e.g., dryness, moisture, wear, or scarring), and subtle variations in finger placement and angle, the minutiae (location and orientation) extracted from two impressions of the same finger are never identical. Traditional exact-match identity-based signature (IBS) schemes require the verification input to be exactly identical to the enrolled template, and thus would reject the second impression due to minor differences, preventing legitimate users from completing payments and severely degrading system usability and user experience. In contrast, a FIBS scheme allows a transaction to be successfully verified as long as the intersection size of the attribute sets, extracted from two impressions of the same finger (obtained by quantized minutiae coordinates and orientations via hash mapping), reaches or exceeds a preset threshold *t*. This error-tolerant mechanism effectively accommodates the inherent noise in biometric acquisition while maintaining sufficient security. Meanwhile, the security of the scheme is based on the Computational Diffie–Hellman (CDH) assumption, ensuring that an adversary cannot forge a valid signature. Moreover, due to the preimage resistance and collision resistance of cryptographic hash functions, the adversary cannot recover the original biometric template. Therefore, FIBS strikes a balance between high availability (tolerating biometric variations) and strong security (forgery resistance and template irreversibility), making it particularly suitable for scenarios such as mobile payments, access control, and remote identity authentication. In 2009, Wang and Kim [9] further refined the notion and security framework of FIBS scheme and proposed two novel constructions: one is based on a certification approach using standard signatures, while the other is a transformation derived from convertible identification schemes under the random oracle model (ROM). In 2011, Yang et al. [10] introduced the first practical FIBS scheme and extended the applicability of their construction to biometric authentication. In 2012, Wang [11] presented a bilinear pairing-based FIBS scheme with provable security, demonstrating existential unforgeability against chosen message and selective fuzzy identity attacks in the ROM under the discrete logarithm assumption. Yao et al. [12] introduced an innovative FIBS scheme based on the short integer solution (SIS) problem in 2014, proving its existential unforgeability against adaptively chosen message and identity attacks. In the same year, Xiong et al. [13] analyzed and improved the existing FIBS scheme to enhance security. In 2016, Wang et al. [14] proposed the concept of fuzzy certificateless signature, addressing the key escrow issue in FIBS while preserving its error tolerance property. In the same year, Islam et al. [15] established the formal and security frameworks for the proposed BIO-IDMS scheme. In 2017, Zhang et al. [16] introduced a FIBS scheme based on lattice cryptography for biometric authentication applications. In 2021, Cao et al. [17] presented a fuzzy identity-based ring signature (LFIBRS) construction that can be applied for the situations requiring the signer’s real identity verification. In the same year, Shan et al. [18] designed two efficient biometric-based signature constructions in the identity-based setting, utilizing bilinear pairings over elliptic curves to improve efficiency and practicality for resource-constrained applications. While attribute-based signatures (ABS) [19,20] generalize identity-based signatures by binding a signature to the satisfaction of a structural access policy over a set of exact-match attributes, fuzzy identity signatures relax the matching rule to a distance-based tolerance (e.g., $|\omega \cap \omega^{\prime }|\;\geq t$), enabling authentication from noisy biometric representations without exposing raw templates. In this view, FIBS can be seen as an IBS instantiation where the “identity” is a fuzzy attribute set, and also as a restricted form of ABS whose policy reduces to a threshold predicate on overlapping attributes.

### 1.2. Motivation and Contributions

In 2022, Qu et al. [21] introduced a FIBS scheme under the standard model (SM). The ROM regards the hash function as a completely random ideal model, and the only way for the attackers to obtain the hash function value is to access the random oracle, as mentioned in the literature [22,23]. In 2022, Wang et al. [22] proposed a novel pairing-free certificateless signature scheme under the ROM by using the discrete logarithm problem based on elliptic curve group. In 2024, Shim [23] pointed out that Wang et al.’s scheme [22] is vulnerable to key recovery attacks and introduced a new pairing-based certificateless signature scheme that is secure under the ROM. Some cryptographic schemes that enjoy provable security in the ROM may become vulnerable when deployed in practical applications. In the SM, the security proof of the cryptographic scheme only relies on some classical difficult problem assumptions and the characteristics of hash function, which can be implemented in reality without involving any ideal assumptions. In the security proof process of SM, the hash function is not regarded as an ideal random function, so the security given in the SM will be more stable and reliable than that given in the ROM. Cryptographic schemes built upon the SM have higher security than those schemes built upon the ROM. It is of great significance to design an efficient cryptographic scheme under the SM, as mentioned in the literature [24,25]. In 2020, Deng et al. [24] constructed a novel certificateless signature scheme along with a novel certificateless aggregate signature (CLAS) scheme under the SM by using only three pairing operations. In 2024, Deng et al. [25] created a novel CLAS scheme suitable for internet of vehicles based on the SM and only two pairing operations. At present, designing provably secure cryptographic schemes under the SM has become a research hotspot in modern cryptography. In this paper, building upon the existing research findings, our contributions are as follows:

- We adopt fingerprint minutiae as the biometric modality and present a feature extraction algorithm E that transforms raw minutiae into quantized, privacy-preserving attribute sets. This algorithm discards the minutia type to enhance robustness, uses a unified parameter *Q*_0_ for spatial and angular quantization, and maps encoded minutiae bitstrings to finite-field elements for lightweight privacy-preserving biometric cryptography. We present a False Rejection Rate (FRR)–False Acceptance Rate (FAR) trade-off framework to calibrate matching threshold *t* using attribute sets intersection cardinality, with adjustable *n* and *Q*_0_ for qualified error performance.
- We introduce a new efficient FIBS scheme under the SM. Our new scheme is proven to be unforgeable in SM for any polynomially bounded adversary under a selective identity attack model. The security model in the schemes [11,14,18] are all ROM, the security model for the scheme [13] remains unspecified, and both [21] and our proposed scheme utilize the SM. Hence, compared to the existing FIBS schemes [11,14,18] based on the ROM, our new FIBS scheme has a strong security model.
- A comparison of the computation and communication cost is shown between the proposed scheme with the existing FIBS scheme in [21] based on the SM. Compared to [21], our scheme features lower sign cost, verification cost and total cost. In the signature verification stage, our new FIBS scheme only requires the operations of two bilinear pairings, while the FIBS scheme [21] based on the SM requires the operations of three bilinear pairings. The private key size in our scheme is identical to that of [21] and our scheme exhibits a smaller size of message and signature than [21]. Compared to the existing FIBS scheme [21] based on the SM, our new FIBS scheme reduces total computation consumption by approximately 33.55% and achieves a significant reduction in communication consumption, saving approximately 68.07% of the message and signature size, which is suitable for biometric authentication.

### 1.3. Roadmap

The paper’s organization is summarized in the following. In Section 2, some preliminaries that contain bilinear pairings, Shamir’s secret sharing, feature extraction algorithm E and determination of threshold *t* and *n* are presented. In Section 3, the system model and security model of FIBS are introduced. We present the description of our scheme in Section 4. Our scheme’s analysis of security and efficiency are presented in Section 5 and Section 6, respectively. In the end, some conclusions are shown in Section 7.

## 2. Preliminaries

This section introduces some preliminaries as follows. The symbols used throughout the paper are provided in Table 1.

### 2.1. Bilinear Pairings

Let $G_{1}=\left(P\right)$ be defined as an additive group with prime order *q*, and $G_{2}$ as a multiplicative group with the same order. Let $e:G_{1}\times G_{1}\to G_{2}$ denote a bilinear map with the following properties.

- Bilinearity: for all $P_{1},P_{2}\in G_{1}$, $a,b\in Z_{q}$, $e\left(aP_{1},bP_{2}\right)=e{\left(P_{1},P_{2}\right)}^{ab}$.
- Non-degeneracy: there exist $P_{1},P_{2}\in G_{1}$ such that $e\left(P_{1},P_{2}\right)\neq 1_{G_{2}}$.
- Computability: an efficient algorithm exists for computing $e(P_{1},P_{2})$ for all $P_{1},P_{2}\in G_{1}\;$.

Computational Diffie–Hellman Problem (CDHP): given the tuple (P,aP,bP), where $P\in G_{1}$ and $a,b\in Z_{q}^{*}$ are both unknown, to compute abP.

Computational Diffie–Hellman Assumption (CDHA): for any probabilistic polynomial-time (PPT) adversary A, the advantage ${\mathrm{Adv}}_{A}^{\mathrm{CDH}}\left(\lambda \right)=\mathrm{Pr}\left[A\left(P,aP,bP\right)=abP|a,b\in Z_{q}^{*}\right]$ of adversary A to solve CDHP is negligible, where λ is the security parameter.

### 2.2. Shamir’s Secret Sharing

Shamir [26] introduced a (t,n) threshold secret sharing scheme for distributing a secret value $s\in Z_{q}^{*}$ (selected by a dealer) among a set $P=\{P_{1},P_{2},\ldots ,P_{n}$} of *n* participants. This scheme ensures that any subset of at least *t* participants can reconstruct the secret value *s*, while a group of fewer than *t* participants learns nothing. To share the secret *s*, the dealer chooses a polynomial $f\left(x\right)\in Z_{q}\left[x\right]$ of degree t−1 randomly such that $f\left(x\right)=s+\sum_{j=1}^{t-1}a_{j}x^{j}$. Each participant $P_{i}$ is assigned a unique public element $\alpha_{i}\in Z_{q}^{*}$, and their secret share is computed as $s_{i}=f\left(\alpha_{i}\right)$. The share $s_{i}$ is then securely transmitted to $P_{i}$. For reconstruction, any qualified subset S⊆P with |S|=t can compute the secret as $s=f\left(0\right)=\sum_{P_{i}\in S}\Delta_{\alpha_{i},S}\left(0\right)s_{i}$, where $\Delta_{\alpha_{i},S}\left(x\right)=\prod_{P_{k}\in S,k\neq i}\frac{x-\alpha_{k}}{\alpha_{i}-\alpha_{k}}\;\mathrm{mod}\;q$ is the Lagrange interpolation coefficient.

### 2.3. Feature Extraction Algorithm E

Our scheme is instantiated with fingerprint minutiae as the biometric modality. Let bio denote a raw grayscale fingerprint image acquired by a sensor. The feature extraction algorithm E is formally defined as $E:\mathrm{bio}⟶\omega =\left\{\omega_{1},\ldots ,\omega_{n}\right\}\subset Z_{q}$, which transforms bio into a set of quantized, privacy-preserving attributes. The algorithm consists of the following steps.

- Preprocessing and Minutiae Detection. The raw image undergoes standard preprocessing (contrast normalization, Gabor filtering, binarization, and thinning) to extract the ridge skeleton. A minutiae detector then locates two types of local ridge singularities: ridge endings and bifurcations. Each detected minutia $m_{i}$ is initially represented by its spatial coordinates $\left(x_{i},y_{i}\right)$ and orientation $\theta_{i}$ (in degrees). To ensure robustness, low-quality or boundary-proximate minutiae are filtered out. Furthermore, to reduce the risk of template reconstruction, the minutia type (ridge ending/bifurcation) is intentionally discarded, retaining only the geometric attributes. This results in a set of *n* reliable minutiae $\left\{m_{1},m_{2},\ldots ,m_{n}\right\}$, where *n* is a system parameter (typical values range from 30 to 80).
- Translation and Rotation Normalization. To achieve invariance against translation and rotation, we define a canonical coordinate system. Let $\left(x_{c},y_{c}\right)$ denote the core point (or centroid) of the fingerprint. Each minutia’s coordinates are translated so that the core point becomes the origin:
  (1) $$x_{i}^{\prime }=x_{i}-x_{c},\;y_{i}^{\prime }=y_{i}-y_{c}.\;$$
  For rotation invariance, we normalize the orientation field. Let $\theta_{\mathrm{ref}}$ be the orientation of the core point (or the mean direction of all detected minutiae). The orientation of the *i*-th minutia is normalized as:
  (2) $$\theta_{i}^{\prime }=\left(\theta_{i}-\theta_{\mathrm{ref}}\right)\;\mathrm{mod}\;360^{\circ }.\;$$
- Spatial and Angular Quantization. We overlay a $Q_{0}\times Q_{0}$ grid on the image plane. The quantized grid indices $\left(r_{i},c_{i}\right)$ for the aligned coordinates $\left(x_{i}^{\prime },y_{i}^{\prime }\right)$ are calculated as:
  (3) $$r_{i}=\left\lfloor \frac{x_{i}^{\prime }}{\Delta }\right\rfloor \;\mathrm{mod}\;Q_{0},\;c_{i}=\left\lfloor \frac{y_{i}^{\prime }}{\Delta }\right\rfloor \;\mathrm{mod}\;Q_{0},\;$$
  where Δ is the width of a single grid cell. Similarly, the normalized orientation $\theta_{i}^{\prime }$ is quantized into *Q*_0_ angular bins:
  (4) $$\beta_{i}=\left\lfloor \frac{\theta_{i}^{\prime }}{360^{\circ }/Q_{0}}\right\rfloor \;\mathrm{mod}\;Q_{0}.\;$$
- Binary Encoding and Hash Mapping. Each triplet $\left(r_{i},c_{i},\beta_{i}\right)$ is converted into a fixed-length binary string by concatenating the binary representations of its components. Specifically, let ${\mathrm{bin}}_{L}\left(\cdot \right)$ denote the binary representation padded to length $L=\lceil \log_{2}Q_{0}\rceil$. The concatenated bitstring for the i-th minutia is $s_{i}={\mathrm{bin}}_{L}\left(r_{i}\right)\left\|{\mathrm{bin}}_{L}\left(c_{i}\right)\right\|{\mathrm{bin}}_{L}\left(\beta_{i}\right)$ of total length 3L bits. Finally, a cryptographic hash function $H:{\left\{0,1\right\}}^{*}\to Z_{q}$ is applied to obtain $\omega_{i}=H\left(s_{i}\right)\in Z_{q}$ for i=1,…,n. Collectively, these values form the attribute set $\omega =\left\{\omega_{1},\omega_{2},\ldots ,\omega_{n}\right\}\subset Z_{q}$, which serves as a compact, noise-tolerant, and privacy-preserving representation of the fingerprint (biometric feature). Note that the minutia type (ridge endings/bifurcations) is not embedded into $s_{i}$; the attribute is formed solely from the quantized location and orientation so that the representation tolerates typical labeling noise in thinned ridge patterns.

Biometric templates privacy preservation: The privacy of the biometric template is preserved by ensuring the stored data is a non-invertible, pseudonymized representation. Specifically, the raw biometric sample (bio) or the skeletonized image is never stored or processed. Instead, the system only retains a set of hash values $\omega =\left\{\omega_{1},\omega_{2},\ldots ,\omega_{n}\right\}\subset Z_{q}$. This is achieved by first quantizing the biometric features (spatial coordinates and orientation) into discrete indices, converting them into binary strings $s_{i}\left(i\in \left\{1,\ldots ,n\right\}\right)$, and then applying a cryptographic hash function $H:{\left\{0,1\right\}}^{*}\to Z_{q}$ to obtain $\omega_{i}=H\left(s_{i}\right)\in Z_{q}$ for i=1,…,n. Due to the preimage resistance and collision resistance of cryptographic hash function, it is computationally infeasible for an attacker to recover the original quantized features or raw biometric data from the stored hash values, or to find a preimage that corresponds to a different biometric sample to launch an impersonation attack. Furthermore, the omission of sensitive metadata, such as minutiae type, reduces the risk of template reconstruction. Thus, the hash values act as “digital pseudonyms”, providing strong privacy guarantees while maintaining the utility for fuzzy matching.

### 2.4. Determination of Threshold t and n

The threshold *t* and *n* are determined based on the biometric error rates (False Rejection Rate (FRR)/False Acceptance Rate (FAR)) through a trade-off analysis. The procedure consists of the following steps:

- Step 1: Data Collection. Multiple impressions of the same finger are collected to form genuine match pairs, and impressions from different fingers are collected to form impostor match pairs. For each pair, the size of the intersection $|\omega \cap \omega^{\prime }|$ of their attribute sets is computed.
- Step 2: Distribution Plotting. The empirical distributions of the intersection sizes are plotted. Genuine matches typically exhibit a large overlap (e.g., 60–90% of common attributes), while impostor matches show a much smaller overlap (e.g., 0–20%).
- Step 3: Selecting *t* for Acceptable Error Rates. The False Rejection Rate (FRR) is the proportion of genuine matches that are incorrectly rejected (i.e., $|\omega \cap \omega^{\prime }|\;<t$). The False Acceptance Rate (FAR) is the proportion of impostor matches that are incorrectly accepted (i.e., $|\omega \cap \omega^{\prime }|\;\geq t$). By adjusting *t*, a balance between FRR and FAR can be achieved. A typical practice is to set *t* such that $\mathrm{FAR}\leq 10^{-6}$ (security requirement) while FRR≤5%(usability requirement). If these constraints cannot be simultaneously satisfied, the parameter *n* (number of minutiae) or the quantization granularity *Q*_0_ should be adjusted to meet security and usability requirements.

## 3. System Model and Security Model of FIBS

### 3.1. System Model of FIBS

**Figure 2:**
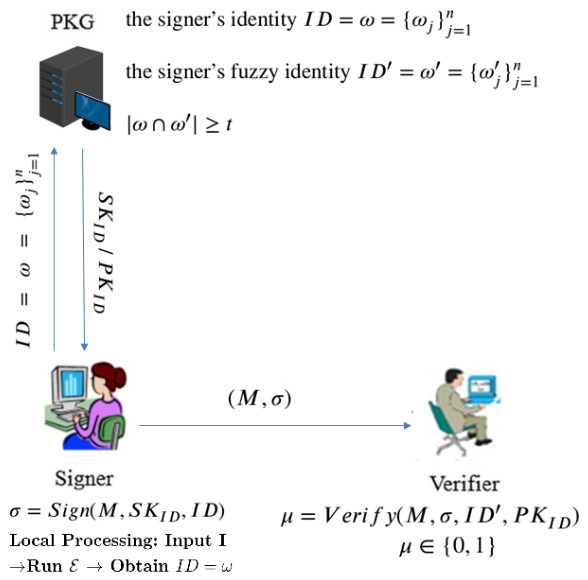
System model of FIBS.

A fuzzy identity-based signature (FIBS) scheme, as presented in Figure 2, comprises three entities: a Private Key Generator (PKG), the signer, and the verifier. The scheme is specified by the following four polynomial-time algorithms:

(1) Setup: Taking λ (the security parameter) as input, the PKG outputs a master private key mk and the system parameters list params, the latter of which includes *t* (an error tolerance parameter).
(2) Key-Extract: This algorithm, executed by the PKG, accepts params, mk, an identity ID represented by an attributes set $\omega ={\left\{\omega_{j}\right\}}_{j=1}^{n}$ (ID $=\omega ={\left\{\omega_{j}\right\}}_{j=1}^{n}$) with each $\omega_{j}\in Z_{q}^{*}$. It generates the corresponding user ID’s private key/public key $SK_{ID}$/${PK}_{ID}$. Note that *t* and *n* are determined based on the biometric error rates (FRR/FAR) through a trade-off analysis in Section 2.4.
(3) Sign: The signer first performs local processing, inputs raw biometric image I to obtain the identity $ID=\omega ={\left\{\omega_{j}\right\}}_{j=1}^{n}$ by running feature extraction algorithm E. Subsequently, this algorithm, executed by the signer, takes as input the identity $ID=\omega ={\left\{\omega_{j}\right\}}_{j=1}^{n}$, the corresponding $SK_{ID}$, a message *M*, and the public system parameter params, and produces a FIBS σ.
(4) Verify: Run by a verifier, this algorithm receives a message–signature pair (M,σ) on a signer’s identity $ID=\omega ={\left\{\omega_{j}\right\}}_{j=1}^{n}$ with his public key $PK_{ID}$, the public system parameters params, and the signer’s fuzzy identity $ID^{\prime }$ represented by an attributes set $\omega^{\prime }={\left\{\omega_{j}^{\prime }\right\}}_{j=1}^{n}$$\left(ID^{\prime }=\omega^{\prime }={\left\{\omega_{j}^{\prime }\right\}}_{j=1}^{n}\right)$ such that $|\omega \cap \omega^{\prime }|\;\geq t$, where each $\omega_{j}^{\prime }\in Z_{q}^{*}$. The algorithm outputs a bit μ∈{0,1}. The FIBS is valid if μ=1, and invalid otherwise.

### 3.2. Security Model of FIBS

The security of a FIBS scheme is defined by the following game involving a challenger C and a polynomially bounded adversary A under the SM. Unlike the game simulation method involved in the proof of security under the ROM, in the security analysis process of SM, the hash function is not regarded as an ideal random function and the security proof in the SM only relies on a classical difficult problem assumption and the characteristics of hash function that can be implemented in reality without involving any ideal assumptions.

Game: The interactive game in which C and A participate is simulated.

Setup: C obtains the params and mk by executing the Setup algorithm, then maintains the mk secret and transmits the params to A. C selects a challenge identity denoted by ${ID}^{*}$ represented by an attributes set $\omega^{*}={\left\{\omega_{j}^{*}\right\}}_{j=1}^{n}$$\left({ID}^{*}=\omega^{*}={\left\{\omega_{j}^{*}\right\}}_{j=1}^{n}\right)$, where $\omega_{j}^{*}\in Z_{q}^{*}$ for j∈{1,…,n}.

Query: A performs the following polynomially bounded number of queries.

- User-Create-Query: A issues this query for an identity $ID=\omega ={\left\{\omega_{j}\right\}}_{j=1}^{n}$. Upon receiving the query, C returns ID’s public key $PK_{ID}$.
- Key-Query: A issues this query for an identity $ID=\omega ={\left\{\omega_{j}\right\}}_{j=1}^{n}$. Upon receiving the query, C returns ID’s private key $SK_{ID}$.
- Sign-Query: When A issues this query for an identity $ID=\omega ={\left\{\omega_{j}\right\}}_{j=1}^{n}$ and a message *M*. Upon receiving the query, C first runs Key-Extract algorithm to generate the private key $SK_{ID}$ and then executes the Sign algorithm to generate the signature σ, which is then sent to A.

Forgery: A gives a forgery tuple $\left(M^{⋄},\sigma^{⋄},ID^{⋄},PK_{ID^{⋄}}\right)$, consisting of a message $M^{⋄}$, a signature $\sigma^{⋄}$, an identity $ID^{⋄}$ corresponding to an attributes set $\omega^{⋄}={\left\{\omega_{j}^{⋄}\right\}}_{j=1}^{n}$$\left(ID^{⋄}=\omega^{⋄}={\left\{\omega_{j}^{⋄}\right\}}_{j=1}^{n}\right)$ and $ID^{⋄}$’s public key $PK_{ID^{⋄}}$, where each $\omega_{j}^{⋄}\in Z_{q}^{*}$, $\sigma^{⋄}$ is associated with $\left(M^{⋄},ID^{*}\right)$. We call that A wins the aforementioned Game, provided that all of the following conditions are met:

(1) A did not issue a Key-Query or a Sign-Query for an identity $ID=\omega ={\left\{\omega_{j}\right\}}_{j=1}^{n}$ such that $|\omega \cap \omega^{*}|\;\geq t$.
(2) $|\omega^{⋄}\cap \omega^{*}|\;\geq t$.
(3) Verify $(params,M^{⋄},\sigma^{⋄},ID^{⋄},PK_{ID^{*}})=1$.

**Definition 1.**  *A FIBS scheme achieves selective identity unforgeability if the success probability $P_{r}\left[A\;wins\right]$ of a polynomially bounded adversary A under a selective identity attack model in the aforementioned game is negligible.*

## 4. Fuzzy Identity-Based Signature Scheme

- Setup: On input λ (the security parameter), let $G_{1}$ and $G_{2}$ be groups with prime order *q* and $e:G_{1}\times G_{1}\to G_{2}$ be a bilinear map. The PKG picks two generators *P*, $Q\in G_{1}$, and PKG works as follows:
  (1) PKG randomly selects an element $x\in Z_{q}^{*}$, which serves as the master secret key mk and sets $P_{pub}=xP$ as the system public key.
  (2) PKG selects an error tolerance parameter *t*, two one-way and collision-resistant cryptographic hash functions $H_{1},H_{2}:{\left\{0,1\right\}}^{*}\to Z_{q}^{*}$ for the system.
  (3) PKG releases $params=\{G_{1},$$G_{2},e,q,P,Q,P_{pub},$$t,H_{1},H_{2}\}$ as the system parameters.
- Key-Extract: On input mk, params together with the user’s identity ID represented by an attributes set $\omega ={\left\{\omega_{j}\right\}}_{j=1}^{n}$, where $ID=\omega ={\left\{\omega_{j}\right\}}_{j=1}^{n}$ and $\omega_{j}\in Z_{q}^{*}$ for j∈{1,…,n}. PKG works as follows.
  (1) PKG chooses $r_{ID}\in Z_{q}^{*}$ randomly, computing $d_{ID}=r_{ID}+q_{ID}x$ and $R_{ID}=r_{ID}P$, where $q_{ID}=H_{1}(ID,$$R_{ID})$.
  (2) PKG randomly chooses a polynomial $q\left(x\right)\in Z_{q}^{*}\left[x\right]$ with degree t−1 such that $q\left(0\right)=d_{ID}$, sets $D_{j}=q\left(\omega_{j}\right)$ for j∈{1,…,n}, then defines $SK_{ID}={\left\{D_{j}\right\}}_{j=1}^{n}$ as the private key corresponding to the user’s identity ID and then transmits $\left(SK_{ID},R_{ID}\right)$ to the user via a secure channel, where $R_{ID}$ is the user’s public key $PK_{ID}$, and *t*, *n* are determined based on the biometric error rates (FRR/FAR) through a trade-off analysis in Section 2.4.
    The user can validate the private key by examining whether the equation $\sum_{\omega_{j}\in I_{t}}\Delta_{\omega_{j},I_{t}}\left(0\right)D_{j}P=R_{ID}+q_{ID}P_{pub}$ holds, where $I_{t}\subseteq \omega$ is a *t*-element subset and $\Delta_{\omega_{j},I_{t}}\left(x\right)=\prod_{\omega_{i}\in I_{t},i\neq j}\frac{x-\omega_{i}}{\omega_{j}-\omega_{i}}\;\mathrm{mod}\;q$. The private key is valid if and only if the equation is satisfied.
- Sign: To produce a signature for a message $M\in {\left\{0,1\right\}}^{*}$, a signer first performs local processing, inputs a raw biometric image I, and runs feature extraction algorithm E to obtain the identity set $ID=\omega ={\left\{\omega_{j}\right\}}_{j=1}^{n}\left(\omega_{j}\in Z_{q}^{*}\right)$. Subsequently, the signer performs the cryptographic signing operations based on ID as follows:
  (1) Randomly chooses $t_{0}\in Z_{q}^{*}$, a polynomial $f\left(x\right)\in Z_{q}\left[x\right]$ with the degree t−1 such that $f\left(0\right)=t_{0}$, to set $T=t_{0}Q$, $t_{j}=f\left(\omega_{j}\right)$ for 1≤j≤n.
  (2) Sets $h=H_{2}\left(ID,R_{ID},T,M,P_{pub},Q\right)$, $V_{j}=t_{j}+hD_{j}$ for j∈{1,…,n}.
  (3) Outputs the signature $\sigma =\{ID,R_{ID},T,V={\left\{V_{j}\right\}}_{j=1}^{n}\}$ of the message *M*.
- Verify: To validate a signature $\sigma =\{ID,R_{ID},T,V={\left\{V_{j}\right\}}_{j=1}^{n}\}$ of a message *M* under the signer’s identity $ID=\omega ={\left\{\omega_{j}\right\}}_{j=1}^{n}\left(\omega_{j}\in Z_{q}^{*}\right)$ with his public key $PK_{ID}=R_{ID}$, given a signer’s fuzzy identity $ID^{\prime }=\omega^{\prime }={\left\{\omega_{j}^{\prime }\right\}}_{j=1}^{n}\left(\omega_{j}^{\prime }\in Z_{q}^{*}\right)$ such that $|\omega \cap \omega^{\prime }|\;\geq t$. The verifier implements the following steps:
  (1) Sets $q_{ID}=H_{1}\left(ID,R_{ID}\right)$ and $h=H_{2}$(ID,$R_{ID},$T,M,$P_{pub},$Q).
  (2) Chooses a random *t*-element subset I from $\omega \cap \omega^{\prime }$, then the signature σ is valid, a bit μ=1 is generated if Equation (5) holds; and σ is invalid, a bit μ=0 is generated if Equation (5) does not hold.
    (5) $$\begin{array}{cc} & e(\sum_{\omega_{j}\in I}\Delta_{\omega_{j},I}\left(0\right)V_{j}Q-T,P)\\ \; & \;=e(h\left(R_{ID}+q_{ID}P_{pub}\right),Q). \end{array}$$

On our scheme’s correctness, we can refer to Equation (6) and we have the following deduction:

(6) $$\begin{array}{cc} \; & e(\sum_{\omega_{j}\in I}\Delta_{\omega_{j},I}\left(0\right)V_{j}Q-T,P)\\ \; & \;=e(\sum_{\omega_{j}\in I}\Delta_{\omega_{j},I}\left(0\right)\left(t_{j}+hD_{j}\right)Q-T,P)\\ \; & \;=e(\sum_{\omega_{j}\in I}\Delta_{\omega_{j},I}\left(0\right)t_{j}Q+\sum_{\omega_{j}\in I}\Delta_{\omega_{j},I}\left(0\right)hD_{j}Q-t_{0}Q,P)\\ \; & \;=e(t_{0}Q+hd_{ID}Q-t_{0}Q,P)\\ \; & \;=e(h\left(r_{ID}+q_{ID}x\right)Q,P)\\ \; & \;=e(h\left(R_{ID}+q_{ID}P_{pub}\right),Q). \end{array}$$

## 5. Security Analysis

This section presents a security proof for the proposed scheme, which is established in the SM by allowing the adversary to compute hash values directly, rather than obtaining them via interactive queries to the challenger.

**Theorem 1.**  *If the CDHA holds, then the new scheme achieves selective identity unforgeability in the standard model against the adversary under the selective identity attack model.*

**Proof.**  Let (P,aP,bP) be a given instance of the CDH problem. The challenger C, aiming to determine abP, executes the Setup algorithm with the security parameter λ and provides the $params=\{G_{1},G_{2},e,q,P,Q=aP,P_{pub}=xP,t,H_{1},H_{2}\}$ to the adversary A. In this setting, both A and C lack knowledge of *a* and *b*, with the critical distinction that C holds the secret value *x*. C selects a challenge identity denoted by ${ID}^{*}=\omega^{*}={\left\{\omega_{j}^{*}\right\}}_{j=1}^{n}$, and each $\omega_{j}^{*}\in Z_{q}^{*}$. A is permitted to make the following queries where in response, C answers them as follows:

- User-Create-Query: C initializes and keeps a list $L_{pub}$ of the tuple (ID,$PK_{ID},$$r_{ID})$. A submits an identity ID, where $ID=\omega ={\left\{\omega_{j}\right\}}_{j=1}^{n}$. If ID is on $L_{pub}$, C returns $\left(ID,PK_{ID},r_{ID}\right)$. Otherwise, for handling a new query on ID, C responds as follows:
  (1) If $|\omega \cap \omega^{*}|\;\geq t$, sets $PK_{ID}=R_{{ID}^{*}}=bP$, and adds (ID,$PK_{ID},$⊥) to list $L_{pub}$.
  (2) If $|\omega \cap \omega^{*}|\;<t$, picks $r_{ID}\in Z_{q}^{*}$ at random, returns $PK_{ID}=R_{ID}=r_{ID}P$, then inserts (ID,$PK_{ID},$$r_{ID})$ to list $L_{pub}$.
- Key-Query: C initializes and keeps a list $L_{K}$ of tuple $\left(ID,SK_{ID}\right)$. When A issues this query for identity $ID=\omega ={\left\{\omega_{j}\right\}}_{j=1}^{n}$, C responds as follows:
  (1) If $|\omega \cap \omega^{*}|\;\geq t$, C fails and terminates the simulation.
  (2) If $|\omega \cap \omega^{*}|\;<t$, C finds $\left(ID,PK_{ID}=R_{ID},r_{ID}\right)$ in $L_{pub}$ and generates ID’s private key $SK_{ID}$ by executing the Key-Extract algorithm, then appends $\left(ID,SK_{ID}\right)$ to the list $L_{K}$, followed by returning $SK_{ID}$ to A.
- Sign-Query: When A makes this query for (M,ID), where *M* is a message and $ID=\omega ={\left\{\omega_{j}\right\}}_{j=1}^{n}$ is the signer’s identity with the public key $PK_{ID}$, C responds as follows:
  (1) If $|\omega \cap \omega^{*}|\;\geq t$, C fails and terminates the simulation.
  (2) If $|\omega \cap \omega^{*}|\;<t$, C finds $\left(ID,PK_{ID}=R_{ID},r_{ID}\right)$ in $L_{pub}$, obtains $SK_{ID}$ by executing the Key-Extract algorithm, and generates $\sigma =\{ID,T,V={\left\{V_{j}\right\}}_{j=1}^{n}\}$ by executing Sign algorithm, followed by returning σ to A.
- Forgery: A outputs a new tuple $(M^{⋄},$$\sigma^{⋄}=\{ID^{⋄},PK_{ID^{⋄}},$$T^{⋄},$$V^{⋄}=$${\left\{V_{k}^{⋄}\right\}}_{k=1}^{n}\})$ that satisfies the conditions of the security model, where $ID^{⋄}=\omega^{⋄}={\left\{\omega_{k}^{⋄}\right\}}_{k=1}^{n}$, $\omega_{k}^{⋄}\in Z_{q}^{*}$ for all k∈{1,…,n}. Since $|\omega^{⋄}\cap \omega^{*}|\;\geq t$, then $PK_{{ID}^{⋄}}=PK_{ID^{*}}=R_{ID^{*}}=bP$; this indicates that $d_{{ID}^{⋄}}=b+q_{{ID}^{⋄}}x$ from the Key-Extract algorithm of our new scheme. Since $\sigma^{⋄}=\left\{ID^{⋄},PK_{ID^{⋄}},T^{⋄},V^{⋄}={\left\{V_{k}^{⋄}\right\}}_{k=1}^{n}\right\}$ is a valid signature, then
  (7) $$\begin{array}{cc} \; & \sum_{\omega_{k}^{⋄}\in I_{t}}\Delta_{\omega_{k}^{⋄},I_{t}}\left(0\right)V_{k}^{⋄}Q-T^{⋄}\\ \; & =\sum_{\omega_{k}^{⋄}\in I_{t}}\Delta_{\omega_{k}^{⋄},I_{t}}\left(0\right)\left(t_{k}^{⋄}+hD_{k}^{⋄}\right)Q-T^{⋄}\\ \; & =\sum_{\omega_{k}^{⋄}\in I_{t}}\Delta_{\omega_{k}^{⋄},I_{t}}\left(0\right)t_{k}^{⋄}Q+\sum_{\omega_{k}^{⋄}\in I_{t}}\Delta_{\omega_{k}^{⋄},I_{t}}\left(0\right)hD_{k}^{⋄}Q-t_{0}^{⋄}Q\\ \; & =t_{0}^{⋄}aP+hd_{{ID}^{⋄}}aP-t_{0}^{⋄}aP\\ \; & =(b+q_{{ID}^{⋄}}x)haP\\ \; & =habP+hq_{{ID}^{⋄}}xaP, \end{array}$$

where $I_{t}\subseteq \omega^{⋄}\cap \omega^{*}$ is a *t*-element subset, $T^{⋄}=t_{0}^{⋄}Q$, $q_{ID^{⋄}}=H_{1}\left({ID}^{⋄},bP\right)$ and $h=H_{2}(ID^{⋄},bP,T^{⋄},M^{⋄},$$P_{pub},aP)$. Hence, C outputs $abP=h^{-1}(\sum_{\omega_{j}^{⋄}\in I_{t}}\Delta_{\omega_{j}^{⋄},I_{t}}$$\left(0\right)V_{j}^{⋄}Q$$-T^{⋄})-q_{{ID}^{⋄}}xaP$ as the solution of CDHP according to Equation (7).

Analysis: we evaluate the probability that the challenger C successfully addresses the given CDHP instance. For this purpose, we examine the following three necessary events that lead to C’s success.

- $Y_{a}$: C completes all key queries without aborting.
- $Y_{b}$: C completes all signing queries without aborting.
- $Y_{c}$: $|\omega^{⋄}\cap \omega^{*}|\;\geq t$.

Let $q_{U},q_{K},q_{S}$ be the maximum numbers of User-Create-Query, Key-Query, Sign-Query, respectively. From the simulation, we know that $P_{r}\left[Y_{a}\wedge Y_{b}\right]=1-\frac{q_{K}+q_{S}}{q_{U}},P_{r}\left[Y_{c}|Y_{a}\wedge Y_{b}\right]=\frac{1}{q_{U}-q_{K}-q_{S}}$. Then, we proceed to evaluate the probability

(8) $$\begin{array}{cc} P_{r}\left[Y_{a}\wedge Y_{b}\wedge Y_{c}\right] & =P_{r}\left[Y_{a}\wedge Y_{b}\right]P_{r}\left[Y_{c}|Y_{a}\wedge Y_{b}\right]=\frac{q_{U}-q_{K}-q_{S}}{q_{U}}\frac{1}{q_{U}-q_{K}-q_{S}}=\frac{1}{q_{U}}. \end{array}\;$$

Consequently, from Equation (8), we conclude that if A has a probability ε in forging a signature, then C solves the CDHP with the probability $\frac{\varepsilon }{q_{U}}$. □

## 6. Comparison with Several Schemes

This section presents a comparative analysis of the proposed scheme against the signature schemes in [11,13,14,18,21] with respect to computation cost, communication cost, and the employed security model. We consider a 512-bit prime *p* and a 160-bit prime *q*. The additive group $G_{1}$ of order *q* is constructed from a point on the super singular elliptic curve $E:y^{2}=x^{3}+1$ over $F_{p}$. A bilinear pairing, $e:G_{1}\times G_{1}\to G_{2}$, is utilized to provide a security level equivalent to that of a 1024-bit RSA algorithm. For clarity, the following notations are used throughout the performance analysis:

$t_{bp}$: Time for a bilinear pairing operation.

$t_{pa}$: Time for an addition in $G_{1}$.

$t_{sm}$: Time for a scalar multiplication in $G_{1}$.

$t_{exp}$: Time for an exponentiation operation in $G_{2}$.

$t_{mul}$: Time for a multiplication in $G_{2}$.

$t_{mtp}$: Time for a map-to-point hash operation.

$t_{h}$: Time for a general hash function evaluation.

The time costs for fundamental operations, as reported by He et al. [27] who implemented them using the MIRACL cryptographic library on a mobile device (Samsung Galaxy S5 with the Google Android 4.4.2 operating system, 2G bytes memory and a Quad-core 2.45G processor), are summarized in Table 2.

The following notation is adopted for element sizes throughout our analysis. Let $\left|G_{1}\right|$, $\left|Z_{q}^{*}\right|$, and |M| represent the bit-lengths of an element in the group $G_{1}$, an element of $Z_{q}^{*}$, and a message *M*, respectively. These are set as $\left|G_{1}\right|$ = 1024 bits and $\left|Z_{q}^{*}\right|$ = 160 bits. Furthermore, the size of a user identity $ID=\omega ={\left\{\omega_{j}\right\}}_{j=1}^{n}$, where each $\omega_{j}\in Z_{q}^{*}$, is given by $\left|ID\right|=n\left|Z_{q}^{*}\right|=160n$ bits. The comparative analysis of the schemes is conducted under the following parameter configuration: |M| = 32 bits, error tolerance parameter d=26 or t=26 and n=30.

A comparative analysis of the computation costs between our scheme and several existing schemes is first presented in Table 3. Based on the data in Table 3, the following observations are made:

(1) For the scheme in [11], the sign cost requires $nt_{pa}+t_{exp}+t_{h}+t_{bp}+2nt_{sm}=841.748$ ms, the verification cost requires $2t_{bp}+t_{mul}+\left(d+1\right)t_{sm}+\left(d-1\right)t_{pa}=429.394$ ms, and the total cost is 113.776+13.486d+26.891n=1271.142 ms;
(2) For the scheme in [13], the sign cost requires $t_{h}+\left(n+1\right)t_{sm}=415.611$ ms, the verification cost requires $d\left(t_{bp}+t_{exp}\right)+t_{sm}+t_{bp}+t_{h}+\left(d-1\right)t_{mul}+t_{pa}=955.467$ ms, and the total cost is 59.708+34.970d+13.405n=1371.078 ms;
(3) For the scheme in [14], the sign cost requires $n\left(3t_{sm}+2t_{pa}\right)+t_{mtp}+t_{sm}=1258.297$ ms, the verification cost requires $t_{mtp}+7t_{bp}+3t_{mul}+2dt_{sm}+2\left(d-1\right)t_{pa}=963.707$ ms, and the total cost is 309.422+26.972d+40.377n=2222.004 ms;
(4) For the scheme in [18]’s BioFIBS, the sign cost requires $n(\left(\left|M\right|+1\right)t_{pa}+2t_{sm})=884.490$ ms, the verification cost requires $\left(\left|M\right|+3\left(d-1\right)\right)t_{pa}+4t_{bp}+2t_{mul}+\left(d+1\right)t_{h}+3dt_{sm}=1186.637$ ms, and the total cost is 133.273+40.514d+29.483n=2071.127;
(5) For the scheme in [18]’s Bio-IBS, the sign cost requires $\left(\left|M\right|+1\right)t_{pa}+2t_{sm}=29.483$ ms, the verification cost requires $\left|M\right|t_{pa}+t_{mul}+t_{mtp}+3t_{bp}=134.321$ ms, and the total cost is 163.804 ms;
(6) For the scheme in [21], the sign cost requires $t_{sm}+t_{h}+n\left(2t_{sm}+t_{pa}\right)=820.191$ ms, the verification cost requires $\left(t+2\right)t_{sm}+t_{mul}+2t_{h}+tt_{pa}+3t_{bp}=475.705$ ms, and the total cost is 138.53+26.891n+13.486t=1295.896 ms;
(7) For our scheme, the sign cost requires $\left(n+1\right)t_{sm}+nt_{pa}+t_{h}=418.041$ ms, the verification cost requires $\left(t+2\right)t_{sm}+\left(t+1\right)t_{pa}+2t_{bp}+2t_{h}=443.065$ ms, and the total cost is 105.89+13.486n+13.486t=861.106 ms.

**Figure 3:**
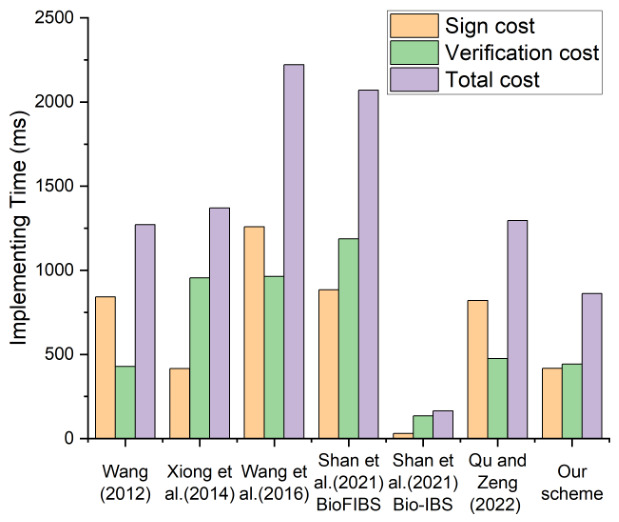
Comparison of computation costs for the schemes of Wang [11], Xiong et al. [13], Wang et al. [14], Shan et al. [18] (BioFIBS and Bio-IBS), Qu and Zeng [21], and our scheme.

The comparative results for the sign cost, the verification cost and the total cost across all schemes are summarized in Table 3 and visually represented in Figure 3. As illustrated in Figure 3, the performance analysis reveals the following: our scheme’s sign cost is lower than those of [11,14,21] and [18]’s BioFIBS, but higher than those of [13] and [18]’s Bio-IBS; our scheme’s verification cost is lower than those of [13,14,21] and [18]’s BioFIBS, but higher than those of [11] and [18]’s Bio-IBS; our scheme’s total cost is lower than those of [11,13,14,21] and [18]’s BioFIBS, but higher than that of [18]’s Bio-IBS. In the signature verification stage, our new FIBS scheme only requires two bilinear pairings, while the FIBS scheme [21] based on the SM requires three. By eliminating one bilinear pairing in this stage, our new FIBS scheme reduces total computation cost by approximately 33.55% compared to scheme [21].

**Figure 4:**
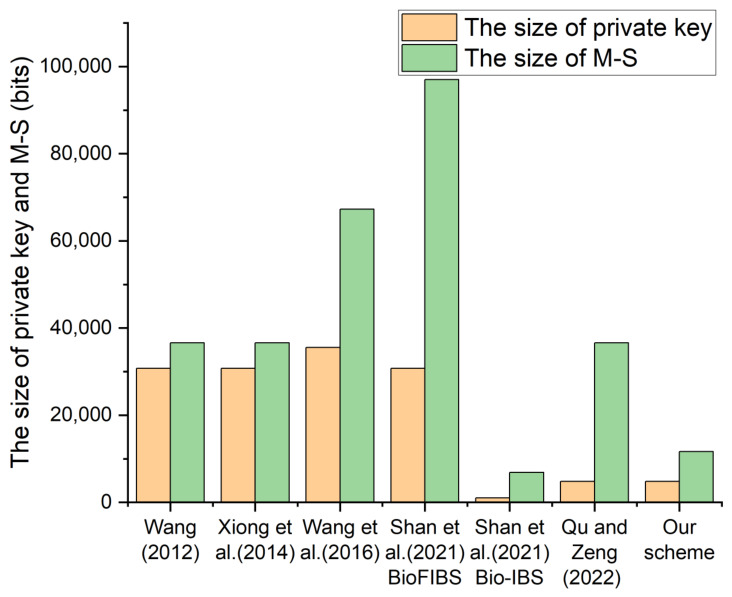
Comparison of communication costs for the schemes of Wang [11], Xiong et al. [13], Wang et al. [14], Shan et al. [18] (BioFIBS and Bio-IBS), Qu and Zeng [21], and our scheme.

In the end, a summary of the communication costs and the security models adopted by each scheme is exhibited in Table 4 and illustrated graphically in Figure 4. According to Table 4, for schemes [11,13], the private key and message and signature sizes are 30,720 bits and 36,576 bits, respectively; for scheme [14], the private key and message and signature sizes are 35,520 bits and 67,296 bits, respectively; for scheme [18]’s BioFIBS, the private key and message and signature sizes are 30,720 bits and 96,992 bits, respectively; for scheme [18]’s Bio-IBS, the private key and message and signature sizes are 1024 bits and 6880 bits, respectively; for scheme [21], the private key and message and signature sizes are 4800 bits and 36,576 bits, respectively; for our scheme, the private key and message and signature sizes are 4800 bits and 11,680 bits, respectively. According to Figure 4, our scheme’s private key size is smaller than the private key sizes of schemes [11,13,14], [18]’s BioFIBS, the same as the private key size of [21] and larger than the private key size of [18]’s Bio-IBS; our scheme’s message and signature size is smaller than the message and signature sizes of [11,13,14,21] and [18]’s BioFIBS, but larger than the message and signature size of [18]’s Bio-IBS. Our scheme achieves a significant reduction in communication cost, saving approximately 68.07% of the message and signature size required by scheme [21]. Additionally, the security model of the schemes [11,14,18] are all ROM, the security model of the scheme [13] is unknown, and the security model of [21] and our scheme are SM. Therefore, according to the above comparative analysis, our scheme demonstrates significant efficiency in terms of computation and communication, has a strong security model, and it is suitable for biometric authentication.

## 7. Conclusions

In the SM, the security proof of the cryptographic scheme only relies on some classical difficult problem assumptions and the characteristics of hash function that can be implemented in reality without involving any ideal assumptions. In the security proof process of SM, the hash function is not regarded as an ideal random function, so the security given in the SM will be more stable and reliable than that given in the ROM. Cryptographic schemes based on the SM have higher security than those schemes based on the ROM. It is of great significance to design a cryptographic scheme under the SM. In this paper, we construct a new FIBS scheme based on SM. Compared with several other schemes in terms of computation, communication, and security model, the results show that our new scheme demonstrates significant efficiency and has a strong security model, and it is suitable for biometric authentication. Another promising direction for future research is the design of a pairing-free fuzzy identity-based signature scheme under the standard model. Eliminating bilinear pairings would significantly improve computational efficiency, particularly on resource-constrained devices such as mobile phones and IoT sensors. However, constructing such a scheme with provable security in the standard model remains a challenging task. We plan to investigate this problem in our future work and aim to develop a lightweight FIBS construction that retains provable security in the standard model without the overhead of bilinear pairings. 

## Figures and Tables

**Table 1:** Symbols and meaning.

Symbols	Meaning
$F_{p}$	A finite prime field.
$G_{1}$	An additive cyclic group of prime order *q*.
$G_{2}$	A multiplicative cyclic group of prime order *q*.
*e*	A bilinear pairings $e:G_{1}\times G_{1}\to G_{2}$.
λ	The security parameter.
$Z_{q}^{*}$	A set of positive integers 1,2,…,q.
*P*, *Q*	Two generators of $G_{1}$.
$P_{pub},x$	The system public key, master secret key, where $P_{pub}=xP$ and $x\in Z_{q}^{*}$.
d,t	Error tolerance parameters.
ID	The identity of a signer, where $ID=\omega ={\left\{\omega_{j}\right\}}_{j=1}^{n}$.
$ID^{\prime }$	The fuzzy identity of the signer with identity ID, where $ID^{\prime }=\omega^{\prime }={\left\{\omega_{j}^{\prime }\right\}}_{j=1}^{n}$ and $|\omega \cap \omega^{\prime }|\geq t$.
$SK_{ID}$	The signer ID’s private key, where $SK_{ID}={\left\{D_{j}\right\}}_{j=1}^{n}$.
$PK_{ID}$	The signer ID’s public key, where $PK_{ID}=R_{ID}$.
$H_{1}$~$H_{2}$	Two secure cryptographic hash functions, where $H_{1}∼H_{2}:{\left\{0,1\right\}}^{*}\to Z_{q}^{*}$.
*M*	A message *M*, where $M\in {\left\{0,1\right\}}^{*}$.
σ	A signature on message *M*.
E	Feature extraction algorithm.

**Table 2:** Cryptographic operation time (milliseconds (ms)).

$t_{\mathrm{bp}}$	$t_{\mathrm{pa}}$	$t_{\mathrm{sm}}$	$t_{\exp}$	$t_{\mathrm{mul}}$	$t_{\mathrm{mtp}}$	$t_{h}$
32.713	0.081	13.405	2.249	0.008	33.582	0.056

**Table 3:** Comparison of computation cost (ms).

Scheme	Sign Cost	Verification Cost	Total Cost
[11]	$nt_{pa}+t_{exp}+t_{h}+t_{bp}+2nt_{sm}=841.748$	$2t_{bp}+t_{mul}+\left(d+1\right)t_{sm}+\left(d-1\right)t_{pa}=429.394$	1271.142
[13]	$t_{h}+\left(n+1\right)t_{sm}=415.611$	$d\left(t_{bp}+t_{exp}\right)+t_{sm}+t_{bp}+t_{h}+\left(d-1\right)t_{mul}+t_{pa}=955.467$	1371.078
[14]	$n\left(3t_{sm}+2t_{pa}\right)+t_{mtp}+t_{sm}=1258.297$	$t_{mtp}+7t_{bp}+3t_{mul}+2dt_{sm}+2\left(d-1\right)t_{pa}=963.707$	2222.004
[18]’s BioFIBS	$n(\left(\left|M\right|+1\right)t_{pa}+2t_{sm})=884.490$	$\left(\left|M\right|+3\left(d-1\right)\right)t_{pa}+4t_{bp}+2t_{mul}+\left(d+1\right)t_{h}+3dt_{sm}=1186.637$	2071.127
[18]’s Bio-IBS	$\left(\left|M\right|+1\right)t_{pa}+2t_{sm}=29.483$	$\left|M\right|t_{pa}+t_{mul}+t_{mtp}+3t_{bp}=134.321$	163.804
[21]	$t_{sm}+t_{h}+n\left(2t_{sm}+t_{pa}\right)=820.191$	$\left(t+2\right)t_{sm}+t_{mul}+2t_{h}+tt_{pa}+3t_{bp}=475.705$	1295.896
Our scheme	$\left(n+1\right)t_{sm}+nt_{pa}+t_{h}=418.041$	$\left(t+2\right)t_{sm}+\left(t+1\right)t_{pa}+2t_{bp}+2t_{h}=443.065$	861.106

**Table 4:** Comparison of communication cost (bits) and security model.

Scheme	The Private Key Size	The Message and Signature (M-S) Size	Security Model
[11]	$n|G_{1}|=30720$	$\left(n+1\right)\left|G_{1}\right|+n\left|Z_{q}^{*}\right|+\left|M\right|=36576$	ROM
[13]	$n|G_{1}|=30720$	$\left(n+1\right)\left|G_{1}\right|+n\left|Z_{q}^{*}\right|+\left|M\right|=36576$	?
[14]	$n(\left|G_{1}\right|+\left|Z_{q}^{*}\right|)=35520$	$\left(2n+1\right)\left|G_{1}\right|+n\left|Z_{q}^{*}\right|+\left|M\right|=67296$	ROM
[18]’s BioFIBS	$n|G_{1}|=30720$	$3n\left|G_{1}\right|+n\left|Z_{q}^{*}\right|+\left|M\right|=96992$	ROM
[18]’s Bio-IBS	$|G_{1}|=1024$	$2\left|G_{1}\right|+n\left|Z_{q}^{*}\right|+\left|M\right|=6880$	ROM
[21]	$n|Z_{q}^{*}|=4800$	$\left(n+1\right)\left|G_{1}\right|+n\left|Z_{q}^{*}\right|+\left|M\right|=36576$	SM
Our scheme	$n|Z_{q}^{*}|=4800$	$2\left|G_{1}\right|+2n\left|Z_{q}^{*}\right|+\left|M\right|=11680$	SM

? indicates unknown (the scheme is neither proven to be secure nor proven to be insecure due to the lack of security proof details), and note that M−S stands for message and signature.

## Data Availability

The original contributions presented in this study are included in the article. Further inquiries can be directed to the corresponding author.
